# Mpox: Neglect has led to a more dangerous virus now spreading across borders, harming and killing people. Leaders must take action to stop mpox now

**DOI:** 10.1371/journal.pgph.0003714

**Published:** 2024-10-17

**Authors:** Christine McNab, Els Torreele, Ayoade Alakija, Aggrey Aluso, Mauricio Cárdenas, Brendan Crabb, Mark Dybul, Patricia J. Garcia, Lawrence O. Gostin, Angel Gurría, Jane Halton, Adam Kamradt-Scott, Michel Kazatchkine, Helena Legido-Quigley, Joanne Liu, Suman Majumdar, Henry E. Mark, Rosemary McCarney, David Miliband, Winnie Mpanju-Shumbusho, Selina Namchee Lo, Anders Nordström, Raj Panjabi, Jorge Saavedra, Nina Schwalbe, Barbara M. Stocking, Eloise Todd, Clare Wenham, Ellen Johnson Sirleaf, Helen Clark

**Affiliations:** 1 Independent Consultant, Toronto, Canada; 2 Former Member of The Independent Panel’s Secretariat, Toronto, Canada; 3 Independent Researcher and Advisor, Geneva, Switzerland; 4 FIND and Special Envoy to ACT-A and Co-Chair of the ACT-A Principals, Abuja, Nigeria; 5 Resilience Action Network Africa, Nairobi, Kenya; 6 Former Minister of Finance of Colombia and Member of The Independent Panel, Bogota Colombia; 7 Burnet Institute, Melbourne, Australia; 8 Diplomat, Physician and Medical Researcher, Member of The Independent Panel, Washington, United States of America; 9 Former Minister of Health of Peru and Member of the PGPHC, Lima, Peru; 10 Professor and Chair in Global Health Law and Member of the PGPHC, Washington, United States of America; 11 Organization for Economic Co-operation and Development, Member of the PGPHC, Mexico City, Mexico; 12 Coalition for Epidemic Preparedness Innovations (CEPI), Member of the PGPHC, Canberra, Australia; 13 Cummings Foundation Professor of One Health Diplomacy, Fletcher School of Law and Diplomacy, Tufts University, Medford, United States of America; 14 The Global Fund to Fight AIDS, Tuberculosis, and Malaria, Member of The Independent Panel, Geneva, Switzerland; 15 Professor in Health Systems, George Institute for Global Health, Imperial College London, London, United Kingdom; 16 Doctors Without Borders, Member of The Independent Panel, Montreal, Canada; 17 Chief Health Officer - COVID and Emergencies, Deputy Program Director, Health Security and Pandemic Preparedness, Burnet Institute, Melbourne, Australia; 18 Independent Consultant, Nottingham, United Kingdom; 19 Former Ambassador and Permanent Representative of Canada to the United Nations, Geneva, Switzerland, Toronto, Canada; 20 Former Foreign Secretary of the United Kingdom, Member of The Independent Panel, New York, United States of America; 21 Former WHO Assistant Director General, Member of the PGPHC, Chêne-Bougeries, Switzerland; 22 Australian Global Health Alliance, Melbourne, Australia; 23 Former Global Health Ambassador for Sweden, Former Head of Secretariat of The Independent Panel, Stockholm, Sweden; 24 Former White House Senior Director for Global Health Security and Biodefense, Former Advisor to The Independent Panel, Springfield, United States of America; 25 AHF Global Public Health Institute, Member of the PGPHC, Miami, United States of America; 26 Spark Street Advisors, New York, United States of America; 27 Murray Edwards College, University of Cambridge, Chair of the PGPHC, Cambridge, United Kingdom; 28 Pandemic Action Network, Brussels, Belgium; 29 Associate Professor of Global Health Policy, Department of Health Policy, London School of Economics and Political Science, London, United Kingdom; 30 Former President of Liberia, Member of The Elders, Co-Chair of The Independent Panel, Monrovia, Liberia; 31 Former Prime Minister of New Zealand, Member of The Elders, Co-Chair of The Independent Panel, Auckland, New Zealand; PLOS: Public Library of Science, UNITED STATES OF AMERICA

*The world cannot continue to simply ‘learn’*, *but not apply the costly lessons of neglecting disease outbreaks.*

The ongoing mpox outbreaks in Africa, involving different clades and including a recently identified clade Ib variant, are of grave concern. Investment is essential now to save lives, protect people, and urgently stop the spread before mpox crosses more borders.

Mpox outbreaks on the African continent had been largely ignored for years. However, when mpox clade IIb unexpectedly spread into wealthy countries just two years ago, the affected countries rapidly invested to contain the epidemic, including with vaccines and experimental treatments, as well as strong community education and mobilization. The ongoing outbreaks in Africa, where mpox had been endemic in several countries, were largely forgotten by the international community [1]. There was hardly any international funding for a response. Vaccines remained out-of-reach and stockpiled in wealthy countries, surveillance and diagnostic capabilities were scant, and treatments were only available to some people, mainly in the context of a clinical trial or compassionate use.

In addition to a growing number of new cases including deaths caused by mpox clade Ia, endemic in the Democratic Republic of the Congo and known to be more virulent than the West-African clade II variant, mpox clade Ib emerged in South Kivu province. This variant is now spreading from person-to-person including through sexual partners and along transport routes and has been detected in at least four more African countries. Together these outbreaks have killed hundreds of children and many adults including people who are immunocompromised, women who are pregnant and their foetuses. Mpox clade IIb is also spreading and has led to the deaths of three men in South Africa [2].

By 16 August 2024, the Africa Centres for Disease Control and Prevention (AfCDC) had reported more than 17,000 total cases of *mpox* clades Ia, Ib and IIb detected in 12 African countries, leading to 517 deaths in 2024 –already more than in all of 2023 [3]. Mpox clade Ia cases are likely vastly under reported. WHO reported in a news conference on 7 August 2024 that 88% of recent deaths linked to clade Ia are children under 15 years of age. The case fatality ratio is 3–4%, and WHO reported it as high as 5% in young children [4, 5].

**In other words, mpox is an ever-growing regional health crisis in Africa, and without urgent action to stop the epidemics when and where they occur, it will continue to spread across borders and continents.** The few tools we have that could help to stop the outbreaks have yet to become adequately available in the most affected low-income countries where they are urgently required, as is financing to support the public health response. Mpox cannot be allowed to continue spreading widely across the African continent or anywhere. The world cannot continue to simply ‘learn’, but not apply the costly lessons of neglecting disease outbreaks.

We welcome the Africa CDC and the WHO’s determination of mpox as both continental and international emergencies [6, 7]. Now the agencies must work closely together, with countries, communities and with partners to ensure a coordinated and effective response. People’s lives cannot wait for red tape or questions of jurisdiction. Most importantly, the emergency declarations must lead to urgent action with adequate financing and technical support such that affected countries can implement optimal public health measures to save lives, protect populations, and limit further spread.

We also call on the WHO and Member States to now apply the spirit of the amended International Health Regulations to the mpox outbreak, which embrace the principles of equity and solidarity. They include an obligation for WHO to assist countries to access health products, strengthen research and development and support local production, for State Parties to help facilitate access to health products, and for financing to be made available [8].

To date several hundred thousand doses of mpox vaccine are publicly reported to have been pledged as donations towards the outbreaks, whereas the Africa CDC had said that at least 10 million doses are required [9–13]. WHO’s move to invite Emergency Use Listing (EUL) of mpox vaccines is a positive step, but could have occurred two years ago. All parties must work efficiently to make the EUL process as short as possible.

More investments are needed into research and development of diagnostics, treatments, and vaccines, with active involvement of African researchers, developers and manufacturers [14]. The commercially available point of care tests seem to have limited utility and independent evidence of their performance is missing, so research is needed to validate and develop better tests that can be scaled up in affected countries [15]. There currently is no prospect for an effective antiviral treatment: the recently released preliminary results of a clinical trial of tecovirimat in clade I in DRC are disappointing, though they did show that supportive care can have an important impact on mortality [16]. While approved vaccines exist their safety and effectiveness need to be confirmed at scale, in different population groups, and in appropriate vaccinations strategies that are adapted to local transmission patterns [15]. Equally important, to ensure long term sustainability and resilience of the response capacity in Africa, technology transfer should be facilitated to enable local or regional production of vaccines closer to where they are needed. Last but not least, medical countermeasures must be developed and made available affordably and equitably to populations in need.

**Overall, attention and action to contain the escalating spread of mpox has been wholly inadequate**. **We urgently call for**:

- Political attention and leadership at national, regional, and global levels to save lives, address gendered impacts, stigmatisation and discrimination and stop the spread.
- For WHO and its Members States to apply the spirit of the amended IHRs to this outbreak and honour the principles of equity and solidarity.
- Millions more dollars in international funding, including from African Union member countries, to be made available to work with communities to contain this outbreak. The lack of funding to date is likely a contributing factor to this expanding outbreak.
- Immediate efforts towards better and more testing, and provision of millions of doses of vaccines at affordable prices for people at risk.
- To this end, GAVI must rapidly deploy the First Response Fund to help purchase vaccines, invest in cold chain and community protection measures [17].
- Effective engagement with and work in partnership with communities to contain this outbreak and leave no one behind—particularly those at risk, and promote prevention measures, vaccine uptake, contact tracing and care to those affected.
- Enhanced surveillance and genomic sequencing to track and understand the spread of this outbreak and better target the response.
- Massively increased investment into the development of better countermeasures and their equitable and affordable access, and support for Africa-based researchers to learn more about all aspects of mpox and develop locally-based and tailored solutions.

## Mpox in Africa can no longer be neglected

During the mpox PHEIC from January of 2022 to May of 2023, mpox clade IIb was detected in 111 countries [18, 19]. Most of the people affected were gay, bisexual and other men who have sex with men, who activated their networks, and together with public health authorities helped to secure community engagement, access to testing, treatment and vaccines and prevent stigmatisation.

When that mpox PHEIC was lifted on May 11 2023 after the surge of cases in wealthy countries subsided, the international response fell flat—even though the IHR Emergency Committee was clearly concerned about the lack of support to stop the outbreaks on the African continent [20]. This lack of attention, leadership, political will and financing to effectively address outbreaks when and where they occur has likely contributed to the worrying increases in cases we are witnessing today.

**Available funding now pales in comparison to what is likely required but is a fraction of the eventual costs of a major outbreak, pandemic or globally endemic disease.** We are encouraged by recent offers of funding however this is not at the scale of what is required [7, 21–23]. WHO and the Africa CDC report that nearly $600 is needed for the first six months alone, and this does not include vaccine costs [24].

Allowing disease to spread and become further endemic has great human and economic costs. The 2014–2016 Ebola outbreak is estimated to have cost $53 billion [25]. The COVID-19 pandemic, which is linked to about 28.5 million deaths of excess mortality, has cost trillions in losses [26, 27]. The impacts continue as COVID again surges around the world, and expensive vaccines and boosters will be needed for years to come.

The risks of new pathogens and spread of existing pathogens to previously non-endemic areas are only growing with climate change and human encroachment on forested areas.

This is why outbreaks must be identified early and stopped in their tracks, and that all regions must have the capacities to research, develop and manufacture locally suitable health products that serve public health and protect their populations [28]. The focus must be on equity and leaving no one behind.

Mpox was neglected, and now it is surging. Leaders must take action and make the investments required to contain mpox and protect people now. It’s beyond time to apply the lessons of HIV, SARS, Ebola and COVID-19 and to stop outbreaks before they spread and can become pandemics.
